# Biosynthesis and Characterization of *Aeonium arboreum*-Derived Silver Nanoparticles: Antimicrobial Activity, Biofilm Inhibition, Antihemolytic Activity, and In Silico Studies

**DOI:** 10.3390/ijms25158039

**Published:** 2024-07-23

**Authors:** Marwah M. Alfeqy, Seham S. El-Hawary, Ali M. El-Halawany, Mohamed A. Rabeh, Saad A. Alshehri, Usama Ramadan Abdelmohsen, Nesreen A. Safwat, Aya M. Serry, Heba A. Fahmy, Marwa I. Ezzat

**Affiliations:** 1 Pharmacognosy Department, Faculty of Pharmacy, Modern University for Technology & Information, Cairo 11571, Egypt; heba.fahmy@pharm.mti.edu.eg; 2 Pharmacognosy Department, Faculty of Pharmacy, Cairo University, Kasr El Aini, Cairo 11562, Egypt; seham.elhawary@pharma.cu.edu.eg (S.S.E.-H.); ali.elhalawany@pharma.cu.edu.eg (A.M.E.-H.); 3 Pharmacognosy Department, College of Pharmacy, King Khalid University, Abha 62251, Saudi Arabia; mrabeh@kku.edu.sa (M.A.R.); salshhri@kku.edu.sa (S.A.A.); 4 Deraya Center for Scientific Research, Deraya University, New Minia 61111, Egypt; usama.ramadan@mu.edu.eg; 5 Pharmacognosy Department, Faculty of Pharmacy, Minia University, Minia 61519, Egypt; 6 Microbiology & Immunology Department, Faculty of Pharmacy, Modern University for Technology & Information, Cairo 11571, Egypt; nesreensafwat@hotmail.com; 7 Pharmaceutical Chemistry Department, Faculty of Pharmacy, Modern University for Technology & Information, Cairo 11571, Egypt; ayaserry@hotmail.com

**Keywords:** *Aeonium arboreum*, nanoparticles, metabolomics, antimicrobial activity, biofilm, docking

## Abstract

Environmentally friendly biosynthesis of silver nanoparticles (AgNPs) from *Aeonium arboreum* (L.) Webb & Berthel is reported for the first time. The synthesized AgNPs were characterized using UV-Vis, FTIR, TEM, Zeta potential, and XRD analysis, revealing high stability (−29.1 mV), spherical shape, and an average size of 100 nm. The antimicrobial activity levels of both *A. arboreum* extract and biosynthesized AgNPs were evaluated against five uropathogens (*Staphylococcus aureus*, *Enterococcus faecalis*, *Escherichia coli*, *Pseudomonas aeruginosa*, and *Candida albicans*). Both the extract and the AgNPs exhibited significant efficacy, particularly against *E. coli*, with inhibition zones of 27 mm and 30 mm, respectively. LC-MS analysis tentatively identified 11 secondary metabolites in the extract, including quercetin-3-O-glucoside, quercetin-3-O-rhamnoside, myricetin 3-glucoside, and daphneresinol. In silico docking studies revealed promising binding affinities of these metabolites in relation to key enzymes involved in bacterial folate synthesis (dihydrofolate reductase (DHFR) and dihydropteroate synthase (DHPS)) and DNA replication (DNA gyrase). These findings demonstrate the potential of *A. arboreum*-based AgNPs and their associated metabolites as a novel therapeutic approach for combating urinary tract infections. Their antimicrobial, antihemolytic, and antibiofilm properties warrant further investigation.

## 1. Introduction

These days, urinary tract infections, or UTIs, are among the most prevalent illnesses. Globally, there has been a rise in antibiotic resistance, which restricts available treatments and can have potentially fatal consequences. The most common causative microorganism for UTIs is *Escherichia coli* (*E. coli*), followed by *Klebsiella pneumoniae*, *Staphylococcus* spp., *Enterococcus faecalis* (*E. faecalis*), *Streptococcus* spp., *Pseudomonas aeruginosa* (*P. aeruginosa*), *Staphylococcus aureus* (*S. aureus*), and *Candida* spp. [1].

Pathogenic bacteria can survive using different virulence factors which help the infection to spread within the host cells. Biofilm formation by microorganisms, which causes UTIs, is an important tool for survival in harsh environments and for the purposes of resisting the immune system and powerful antimicrobials [2]. Furthermore, the hemolytic capability is a typical virulence-associated phenotype of bacteria that is employed to disrupt membranes, lyse cells, and destroy tissues in order to supply the nutrients and iron needed to produce toxins [3]. Thus, biofilm eradication and erythrocyte hemolysis inhibition are significant for therapeutic and infection-control perspectives, as well as the task of overcoming bacterial resistance. Furthermore, the proliferation of Gram-positive bacteria and the associated development of biofilms depend on the ATP-dependent enzyme DNA gyrase, which is categorized as topoisomerase II [4]. On the other hand, the activity of the enzyme dihydrofolate reductase (DHFR), which converts dihydrofolate to tetrahydrofolate (THF), is crucial for controlling the metabolism of folate and overcoming the resistance against *Staphylococcus aureus* (*S. aureus*) and *Escherichia coli* (*E. coli*) [5]. Over seven decades, DHPS has been a proven therapeutic target with a strong history of sulfonamide medication inhibition. However, the use of these medications has been seriously hampered by adverse responses to the sulfonamide class of medicines, in addition to the broad tolerance to these treatments [6].

Nano-biotechnology is an emerging research field that has significantly contributed to various facets of human lives [7]. Silver nanoparticles (AgNPs) are highly efficient, and accounted the best antimicrobial agents for metal nanoparticles [8]. The usage of silver nanoparticles in industrial applications and the medical field is very common because of their antibacterial capabilities [9,10,11,12]. The application of green silver nanoparticles as an antimicrobial drug against bacterial biofilm has been the focus of extensive research [13]. There is a significant amount of interest in finding effective and safe alternatives to traditional treatments, e.g., antibiotics and chemical preservatives, due to growing biofilm resistance, in addition to their adverse outcomes [14]. Gram-negative and Gram-positive bacteria are both strongly inhibited by the biosynthesized nano-sized silver particles. The production of free radicals, which makes it easier to induce membrane-damaging molecules in microorganisms, is one of the mechanisms of Ag-NPs [15,16]. Metal nanoparticles have been prepared using a variety of physicochemical methods [17]. However, the search for economical and ecologically friendly methods has prompted researchers to investigate green approaches for the manufacture of nanoparticles. In this context, utilizing plant extracts as stabilizing/reducing agents for the green synthesis of metal nanoparticles e.g., silver nanoparticles (AgNPs) has gained wide recognition. Medicinal plant extracts are frequently employed as stabilizing/reducing agents in the environmentally friendly production of AgNPs. Many bioactive compounds in the plant, e.g., phenolic acids, flavonoids, alkaloids, and terpenoids, are helpful for the synthesis of silver nanoparticles and reduce silver ions (Ag^+^) for the synthesis of biomolecule-encapsulated AgNPs [18]. Owing to their speedy production rates and the variety of nanoparticle sizes and forms, plants are regarded as the best option for large-scale nanoparticle synthesis [19].

*Aeonium arboreum* (L.) Webb & Berthel is a succulent member of the family Crassulaceae. It has long been used to treat a wide range of illnesses in conventional medical procedures [20]. It has been applied in varying contexts, given its anti-inflammatory, anti-hemorrhoidal, antipyretic, febrifuge, diuretic, and antihypertensive effects [21]. Only a few research efforts have focused on the chemical and biological profile of the *Aeonium* genus and demonstrated its antioxidant and antimicrobial activities [22].

In an attempt to find a natural alternative in the context of antimicrobial resistance, this research effort aimed to ascertain the antimicrobial, antibiofilm, and antihemolytic potential of *A. arboreum* extract and its nanoform product (AgNPs) against some selected microorganisms which cause UTIs. Furthermore, the investigation of extracts’ inhibitory effects on essential bacterial enzymes could be considered in light of a new therapy for UTI, in that they might be used as antibiotic adjuvants. Additionally, the phytochemical composition of the plant was investigated.

## 2. Results and Discussion

### 2.1. Metabolomics Analysis of the Crude Extract of Aeonium Arboreum’s Aerial Parts

**Figure 1:**
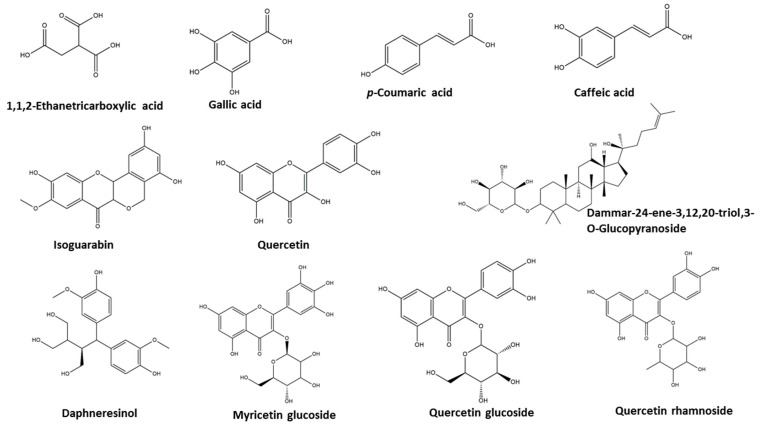
Tentatively identified compounds from UPLC-Q/TOF-MS analysis of methanolic extract of *A. arboreum*’s aerial parts.

Metabolic profiling of the methanol extract of *A. arboreum*’s aerial parts was investigated using reversed-phase ultra-performance liquid chromatography coupled with high resolution mass spectrometry (UPLC–PDA–ESI–qTOF-MS). Eleven different compounds were dereplicated using the Dictionary of Natural Products (DNP) and METLIN databases. They belong to different classes, viz., phenolic acids, flavonoids, sterols, and triterpenes, as shown in Table S1 and Figure 1 and Figure S1. The negative and positive modes revealed the presence of quercetin 3-glucoside, myricetin 3-glucoside, and 1,1,2-Ethanetricarboxylic acid, which were detected in [22], while quercetin-3-*O*-rhamnoside was first isolated in [23], and quercetin, gallic acid, caffeic acid, isoguarabin, daphneresinol, and dammar-24-ene-3,12,20-triol,3-O-glucopyranoside were identified for the first time in *A. arboreum* in the current study.

### 2.2. Characterization of Silver Nanoparticles

#### 2.2.1. UV Analysis

**Figure 2:**
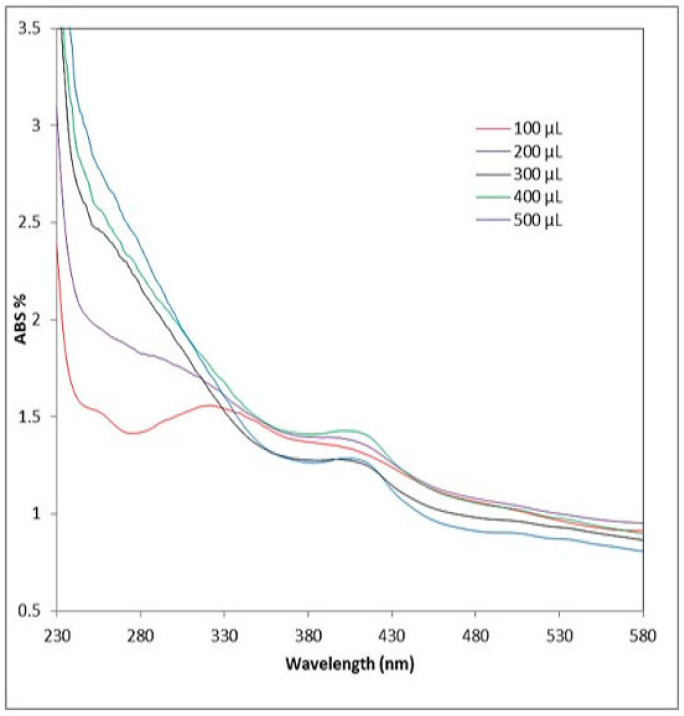
AgNPs’ SPR bands (max) as a function of different extract additions, as determined by UV-vis spectra. ABS, absorption.

The first demonstration of nanoparticle synthesis was performed via UV-Vis spectroscopy because of its selectivity for generated nanoparticles. A yellowish-brown color appeared in the reaction mixture due to the surface plasmon resonance (SPR), confirming the formation of Ag nanoparticles. The SPR band causes the produced samples to absorb in the visible range between 407 and 418 nm, as shown in Figure 2.

#### 2.2.2. FTIR Analysis of AgNPs and *A. arboreum* Extract

**Figure 3:**
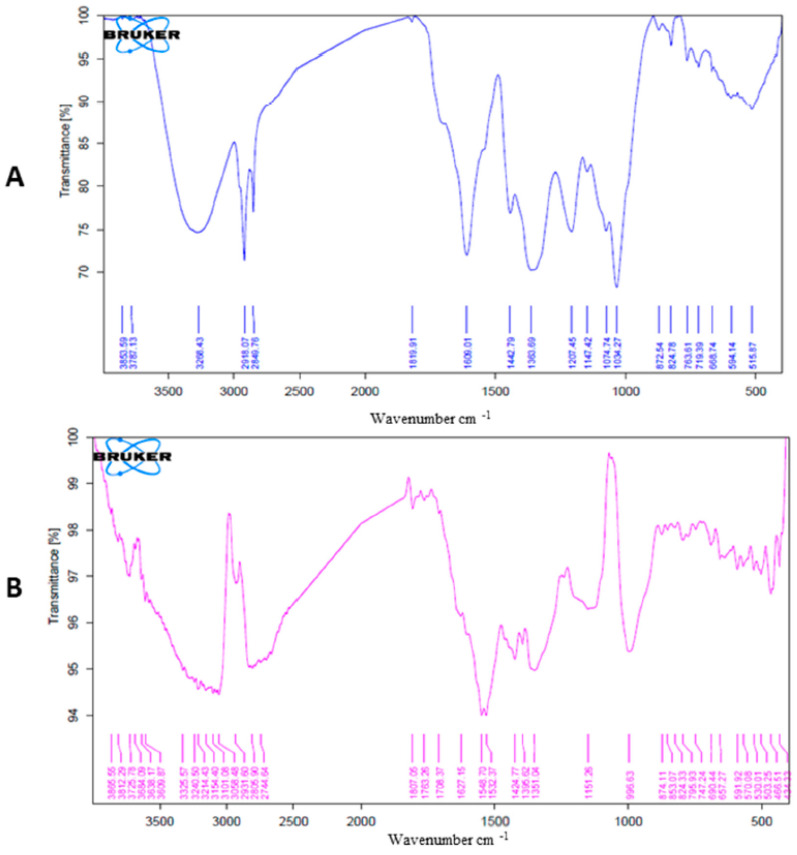
(**A**) *A. arboreum* total methanolic extract’s FTIR spectra, (**B**) *A. arboreum*-prepared AgNPs’ FTIR spectra. (Blue colored lines refers to extract while red refers to synthesized AgNPs).

FTIR spectra of the developed AgNPs and the entire methanolic extract of *A. arboreum* were examined in the 400–4000 cm^−1^ wavelength range to identify the various implicated functional groups. The OH group of the water is adsorbed on the surface of the AgNPs and can be seen as a distinctive absorption band at 3325 cm^−1^ in the AgNPs spectra in Figure 3B The C-H stretching alkane group is demonstrated by bands at 2931 and 2805 cm^−1^. The bands at 1151 and 996 cm^−1^ denoted C-O of ethers, alcohols, or carboxylic acid esters. The appearance of a band at 874.11 demonstrates that the AgNPs experienced C-H bending, and 466.51 was chosen as the location of the distinctive band for the AgNPs’ stretching mode. A carboxylic OH, phenolic, or alcoholic hydroxyl-OH group was present, as shown by the significant broadband peak at 3268 cm^−1^ in the methanolic extract of *A. arboreum* Figure 3A. The C=O (carboxylic and ketonic) absorption band was evident at a lower wavenumber of 1609 cm^−1^. Peaks at 2918 and 2849 cm^−1^ were attributed to the alkane group’s C-H stretching. Bands at 1609–1363 cm^−1^ were attributed to polyphenolic compounds that were stretched either C=C or C-C.

#### 2.2.3. Zeta Potential and Light Scattering Dynamics

**Figure 4:**
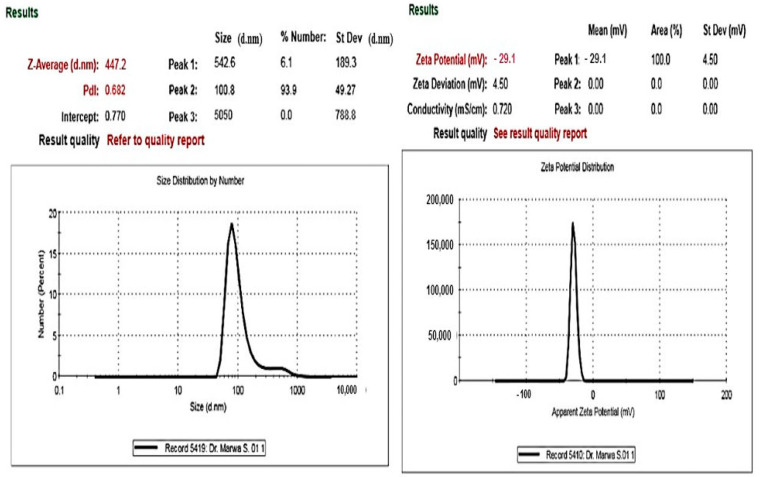
Images of the prepared AgNPs’ size distribution and electrophoretic mobility.

In addition to particle size (PS), the Zeta potential (ZP) value is an essential feature of nanoparticles because it can demonstrate the stability of nanoparticle preparations [24,25]. Higher absolute ZP values can enhance the stability of the system due to strong electrostatic repulsion between the particles, which also inhibits their aggregation. The biosynthesized Ag-nano particles were found to be negatively charged and equally spread throughout the medium, as revealed by the peaking of the signal at −29.1 mV (Figure 4). Also, using the light Scattering Dynamics technique, the metallic shell of the biosynthesized AgNPs was measured; their size was 447.2 nm, on average.

#### 2.2.4. Transmission Electron Microscopy (TEM)

**Figure 5:**
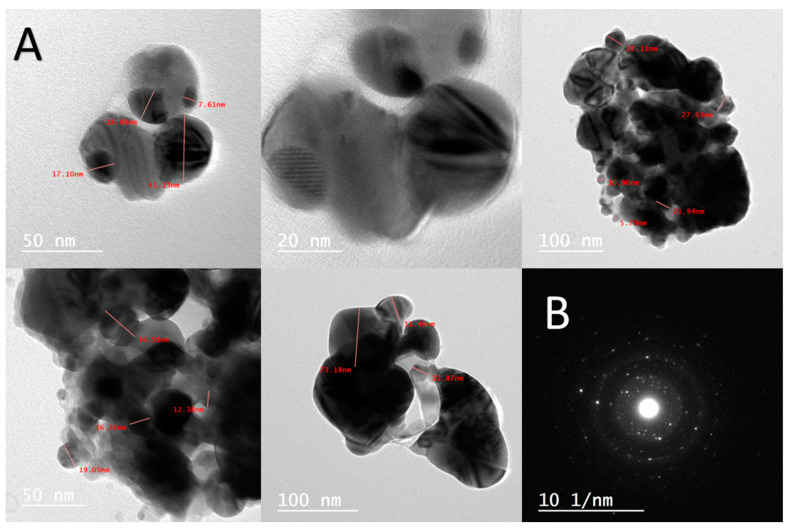
(**A**) Images showing the sizes and shapes of the HRTEM. (**B**) Image of the AgNPs’ selected area electron diffraction (SAED).

TEM was utilized to understand the crystallinity and nature of AgNPs, demonstrating the production of AgNPs in a spherical form, with an average size of 50 nm and a particle size range of 7.61–100 nm (Figure 5A). Additionally, the crystalline form of the LC-AgNPs was validated by means of the selected area electron diffraction (SAED) pattern (Figure 5B).

#### 2.2.5. X-ray Diffraction Analysis (XRD)

**Figure 6:**
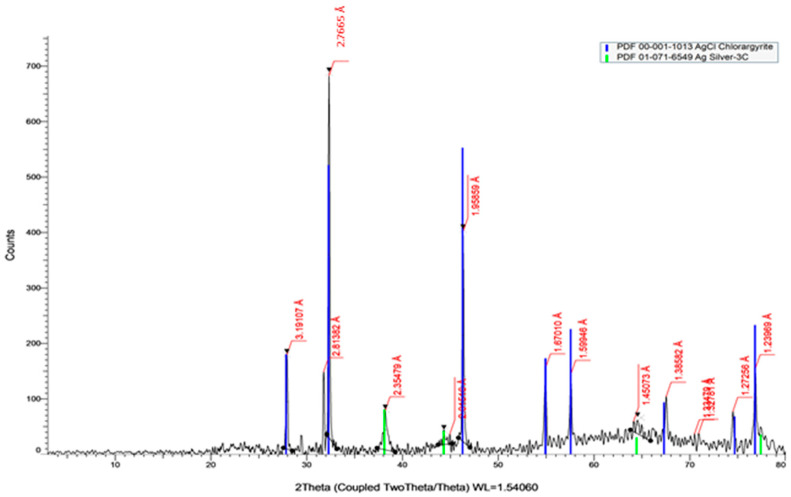
X-ray diffraction (XRD) of synthesized AgNPs of *A. arboreum*.

AgNPs’ structural characteristics were examined using X-ray diffraction (XRD). Bio-synthesized AgNPs of *A. arboreum* extract showed peaks at 2.35 Å, 2.01 Å, 1.45 Å, and 1.23 Å, respectively, corresponding to (38°), (44°), (64°), and (77°), as displayed in Figure 6.

This is the first green synthesis of AgNPs using *A. arboreum* extract, and the UV, FTIR analysis, Zeta-sizer measurements, TEM, and XRD were assessed in order to ensure the proper particle size (100 nm) and the stability (−29 mv) of the synthesized nanoparticles.

### 2.3. Antimicrobial Activity

#### 2.3.1. Antimicrobial Activity of Plant Extract and Its Nanoform

**Figure 7:**
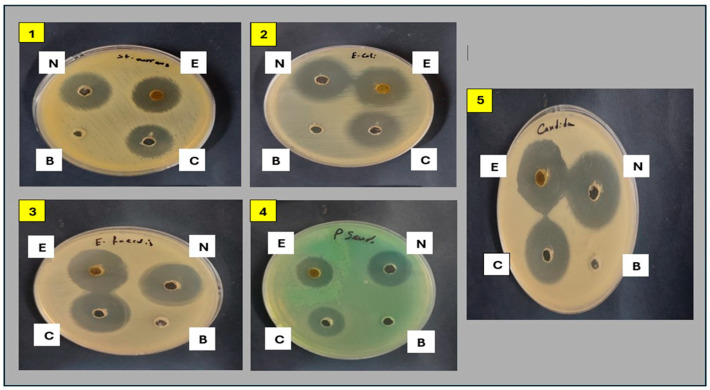
Antimicrobial activity of *A. arboreum* extract and its nanoform (AgNPs) against the strains: (**1**) *S. aureus*, (**2**) *E. coli*, (**3**) *E faecalis*, (**4**) *P. aeruginosa*, (**5**) *C. albicans* using agar well diffusion method.

The antimicrobial activity was evaluated using the agar well diffusion method. The results revealed that the methanolic extract of *A. arboreum* exhibited potential activity against *E. coli*, *E. faecalis*, and *C. albicans*, for each of which the inhibition zones were larger than the reference antibiotic Gentamycin (27 mm, 27 mm, and 28 mm, respectively). Moreover, the plant extract showed good antimicrobial activity against *S. aureus* and *P. aeruginosa*, as recorded in Table 1. The nanoform (AgNPs) was highly effective against all tested microbial strains, for each of which it showed larger inhibition zones than did the methanolic extract of *A. arboreum*. The inhibition zones produced by the nanoform (AgNPs) were also large in comparison to those for gentamycin against the tested microbial strains (Table 1, Figure 7).

#### 2.3.2. Determination of Minimum Inhibitory Concentration (MIC) and Minimum Bactericidal Concentration (MBC)

The methanolic extract of *A. arboreum* showed (MIC) values ranging from 7.8 to 32.25 μg/mL against different species of tested microorganisms. The MIC and MBC results confirmed the higher activity of the AgNPs, as compared to the methanolic extract of *A. arboreum*, against all the tested microorganisms. The AgNPs were highly effective against *P. aeruginosa* and *candida*, with MIC values similar to those achieved by the reference drug gentamycin (31.25 μg/mL and 15.62 μg/mL, respectively). Moreover, the nano-formula inhibited the growth of *S. aureus*, *E. coli*, and *E. faecalis* at MIC values lower than those of gentamycin. The MBC/MIC ratios of *A. arboreum* and AgNPs against all of the tested microorganisms were ≤ 4. Thus, they were considered bactericidal rather than bacteriostatic, as recorded in Table 2.

#### 2.3.3. Determination of Biofilm Inhibition Activity

**Figure 8:**
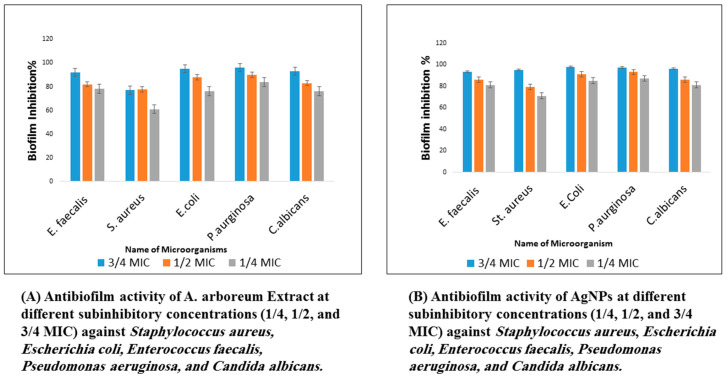
Antibiofilm activity of *A. arboreum* extract (**A**) and AgNPs (**B**) at different subinhibitory concentrations (1/4, 1/2, and 3/4 MIC) against *Staphylococcus aureus*, *Escherichia coli*, *Enterococcus faecalis*, *Pseudomonas aeruginosa*, and *Candida albicans*. The error bars representing the standard errors that indicating low variability of biofilm inhibition percentage values.

**Figure 9:**
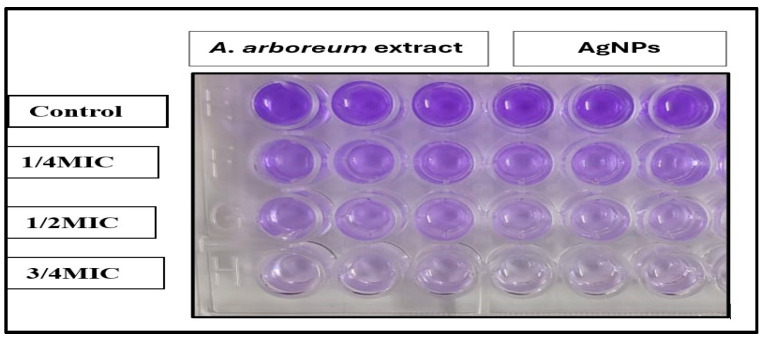
Biofilm inhibition of *S. aureus* by different sub-inhibitory concentrations of *A. arboreum* extract and AgNPs using crystal violet analysis.

The results showed the potential effects of *A. arboreum* extract on the inhibition of biofilm formation against the tested microorganisms at sub-inhibitory concentrations (1/4 MIC, 1/2 MIC, and 3/4 MIC), as demonstrated in (Figure 8). The highest biofilm inhibition of the plant extract was against *E. coli* and *P. aeruginosa*, ranging from 76% to 95% and 84% to 96%, respectively. The *S. aureus* biofilm formation was more efficiently inhibited by AgNPs (95%), as compared to the *A. arboreum* extract (77%), at 3/4 MIC. The nano-formula AgNPs showed higher biofilm inhibition, ranging from 71% to 78% against all tested *microorganisms*, compared to *A. arboreum* extract at a concentration as low as 1/4 MIC (Figure 9). Nevertheless, neither the extracts nor the nanoform could inhibit biofilm formation completely.

#### 2.3.4. Hemolysis Inhibition Activity

**Figure 10:**
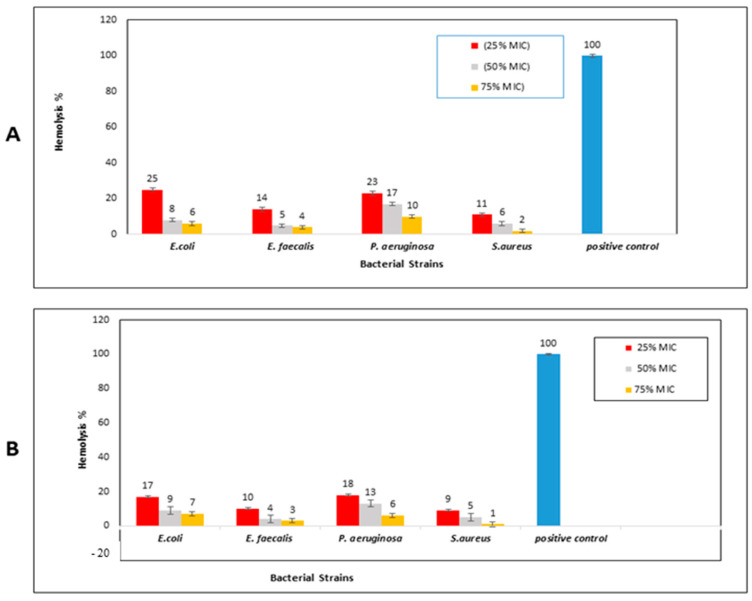
(**A**) Antihemolytic activity of *A. arboreum* extract at different subinhibitory concentrations (1/4, 1/2, and 3/4 MIC) against *Escherichia coli*, *Enterococcus faecalis*, *Pseudomonas aeruginosa*, and *Staphylococcus aureus*, (**B**) Antihemolytic activity of AgNPs at different subinhibitory concentrations (1/4, 1/2, and 3/4 MIC) against *Escherichia coli* (ATCC 8739), *Enterococcus faecalis* (*ATCC 10541*), *Pseudomonas aeruginosa* (ATCC 90274), and *Staphylococcus aureus* (ATCC 6538).

**Figure 11:**
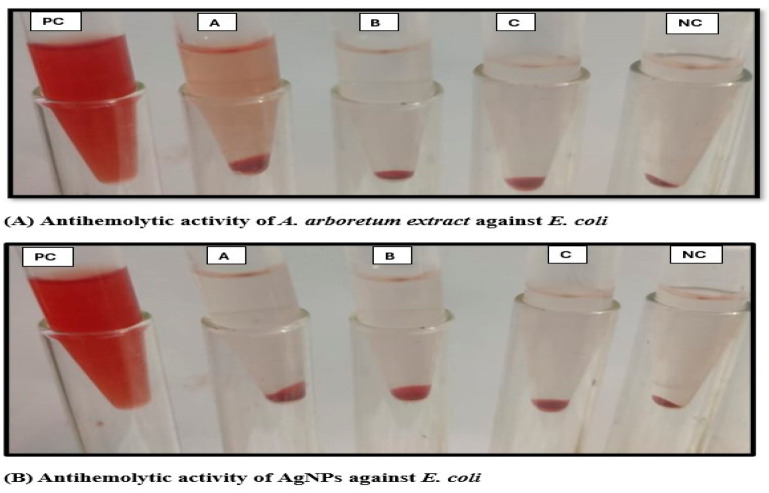
Antihemolytic activity of *A. arboreum* extract (**A**) and AgNPs (**B**) at different sub-inhibitory concentrations against *Escherichia coli*, where A: 1/4 MIC, B: 1/2 MIC, C: 3/4 MIC, PC: Positive Control, and NC: Negative Control).

At subinhibitory concentrations, the plant extract showed potential activity against erythrocyte hemolysis associated with the tested strains of *S. aureus* (2–11%) and *E. faecalis* (4–14%). Although erythrocyte hemolysis produced by *E. coli* and *P. aeruginosa* was partially inhibited by *A. arboreum* extract at 1.9 μg/mL and 7.8 μg/mL, respectively (25% MIC), the antihemolytic activity increased by using 75% of MIC, reaching hemolysis levels as low as 6% and 10%, respectively (Figure 10A and Figure 11).

The results showed a notable increase of nano-formula (AgNPs) antihemolytic activity at a low concentration (25% MIC), compared to that of *A. arboreum* extract, for which it decreased the hemolysis percentages produced by *E. coli* and *P. aeruginosa* to 17% and 18%, respectively. The AgNPs almost prevented hemolysis by *S. aureus* and *E. faecalis* at a concentration of 75% MIC (2% and 3%, respectively), as shown in (Figure 10B and Figure 11).

### 2.4. Molecular Docking

#### Simulations of Molecular Docking

Affinity testing for 11 extracted compounds was performed on three protein targets to investigate the mechanisms of their antibacterial activities. These targets were dihydrofolate reductase (DHFR), (PDB ID: 3frb), dihydropteroate synthase (DHPS), (PDB ID: 3tye), and DNA gyrase, (PDB ID: 4uro). DHFR and DHPS were chosen for the docking studies, as they are two of the most important drug targets for antibacterial activities. When DHFR and DHPS are inhibited, bacterial folic acid fails to be synthesized, leading to false nucleotide formation [26]. In addition, bacterial DNA gyrase, a type IIA DNA topoisomerase that plays an essential role in bacterial DNA replication and transcription, is a clinically validated target for discovering and developing new antibiotics [27].

**Figure 12:**
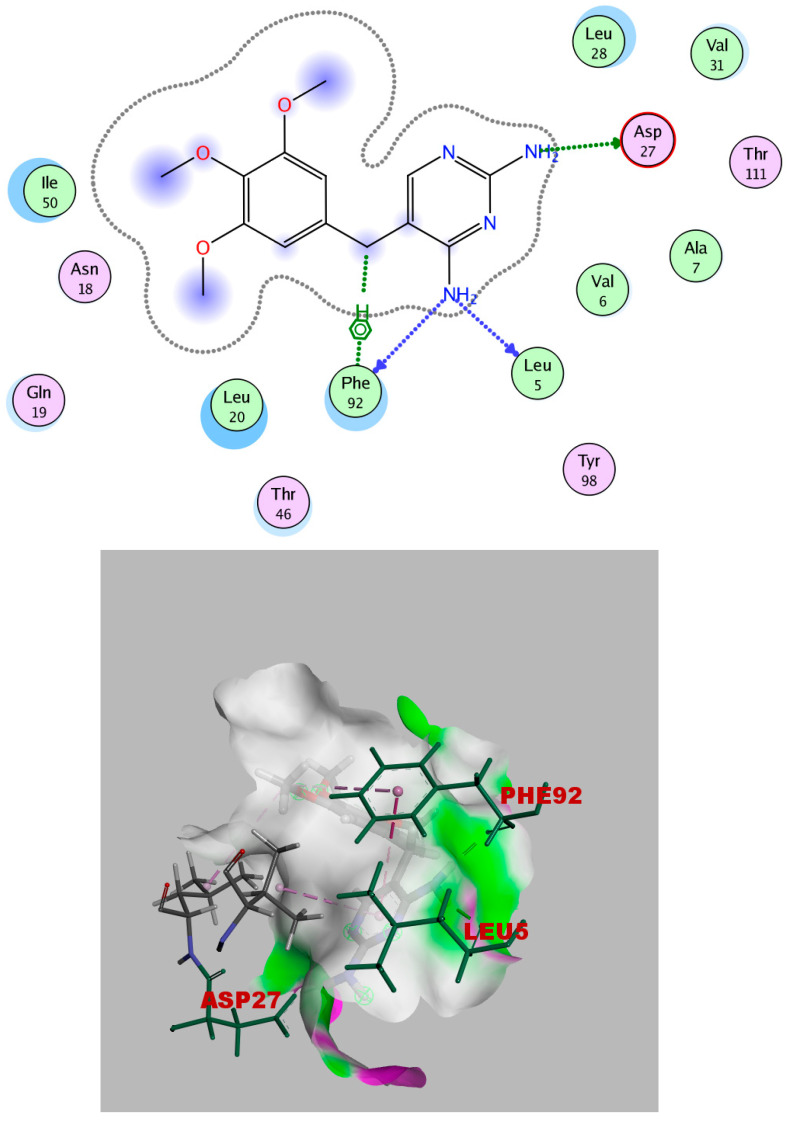
TMP’s 2D and 3D interactions with DHFR.

The crystal structure of *S. aureus* F98Y DHFR complexed with trimethoprim (TMP) was uploaded from the protein data bank (PDB ID: 3frb) www.rcsb.org (accessed on 15 September 2023). In the X-ray structure, the co-crystallized ligand, TMP, and the practical interaction between trimethoprim (TMP) and the wild-type *S. aureus* enzyme demonstrated that the 2,4-diamino pyrimidine (DAP) moiety interacts with Asp27’s side chain through an ionic connection between DAP’s 2-amino group and Asp27. Leu5’s carbonyl oxygen and Phe92 at 2.82 Å are both within hydrogen bonding distance of the amino group at position 4 (Figure 12).

**Figure 13:**
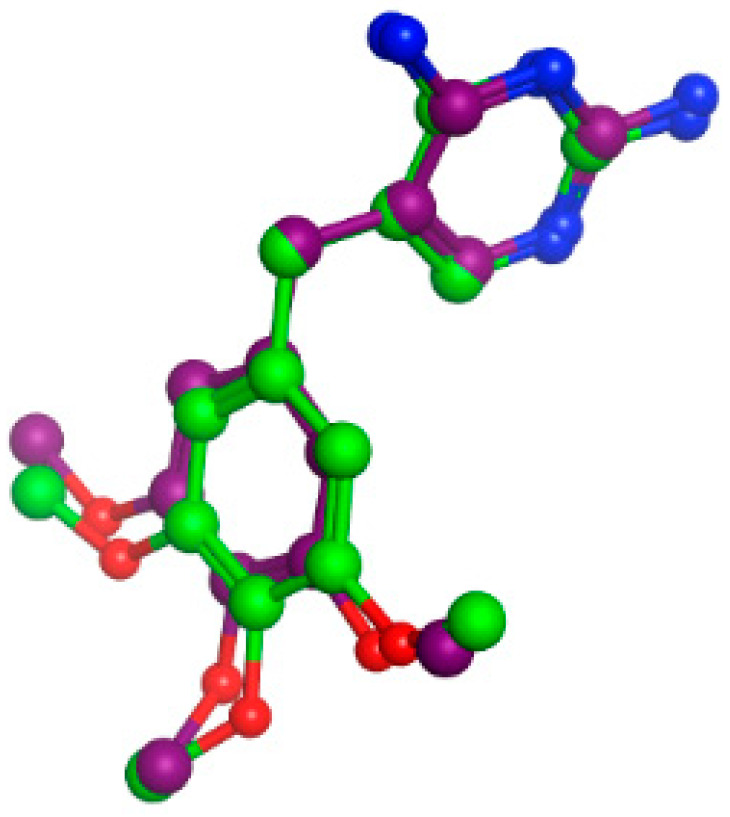
Redocking of STZ (purple) and its superimposition on the co-crystallized STZ (green).

Redocking TMP into the DHFR substrate-binding site was performed through the rigid docking protocol. Findings from the TMP redocking analysis revealed a binding score of (−8.6 kCal/mol) and an RMSD (root mean square deviation) of (1.0105 Å) relative to the co-crystalline ligand; this indicates a well-matched superimposition (Figure 13). The accepted docking technique was proven to be legitimate because it displayed RMSD values below 2.0, and these validation findings demonstrated that the selected docking parameters and methodologies were the best for choosing the ideal docking pose.

On the other hand, the molecular docking analysis of 11 compounds from the extract of *A. arboreum* was docked on DHFR, DHPS, and DNA gyrase active sites, and the following results were obtained, as listed in Tables S2, S3 and S4, respectively.

**Figure 14:**
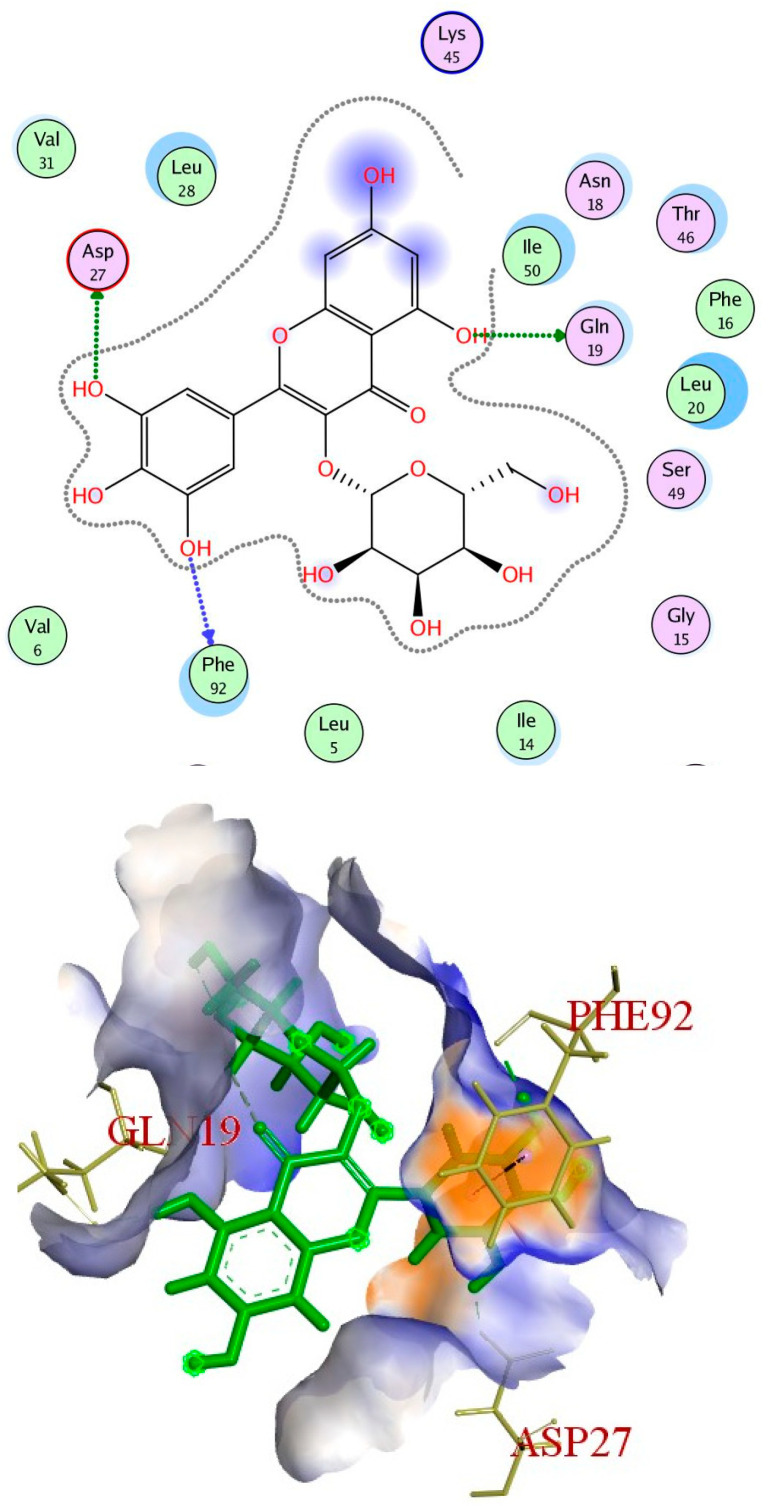
Myricetin-3-glucoside’s 2D and 3D interactions with DHFR.

**Figure 15:**
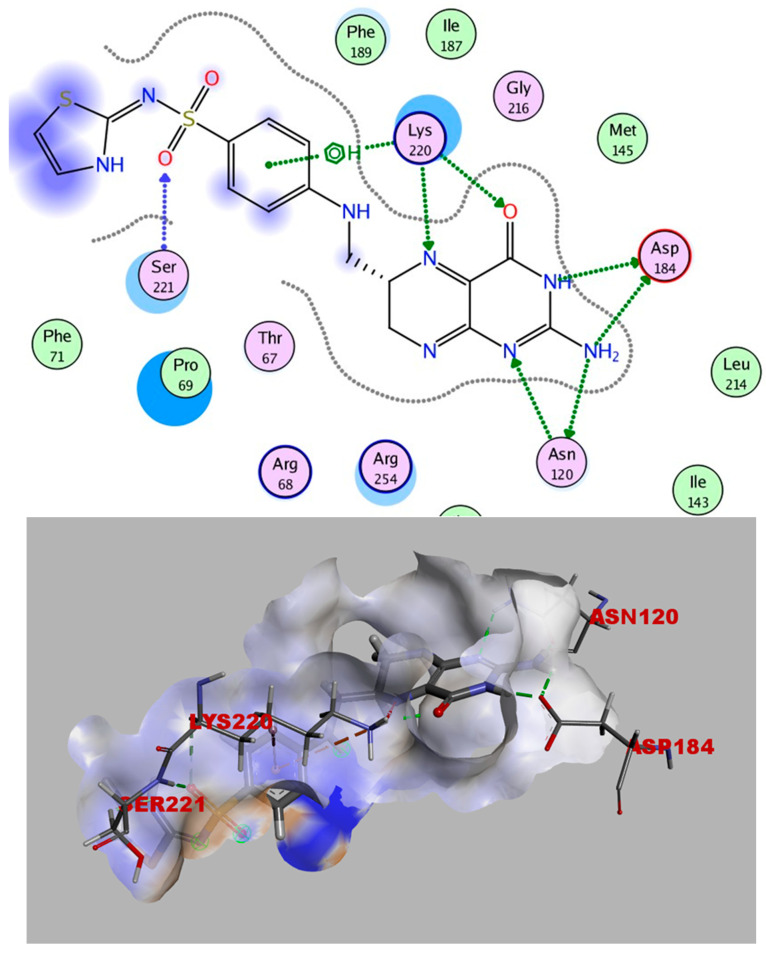
STZ’s 2D and 3D interactions with DHPS.

It is obvious from the results of docking that myricetin-3-glucoside had the best binding energy (−10.723), which exceeded that of the co-crystallized ligand (TMP). Myricetin-3-glucoside, showed common interactions with the amino acids Asp27 and Phe92, in addition to another hydrogen bond with Gln19. The 2D and 3D interactions of myricetin-3-glucoside with the DHFR enzyme are depicted in Figure 14. The crystal structure of dihydropteroate synthase (DHPS) complexed with Sulfathiazole STZ was uploaded from the protein data bank (PDB ID: 3tye) www.rcsb.org (accessed on 15 September 2023). The co-crystallized ligand STZ has four hydrogen-bond acceptors (Asn120, Lys220, and Ser221), three hydrogen-bond donors (Asn120 and Asp184), and one arene-H interaction (Lye220) within the DHPS pockets, according to the docking data in the X-ray structure (Figure 15).

**Figure 16:**
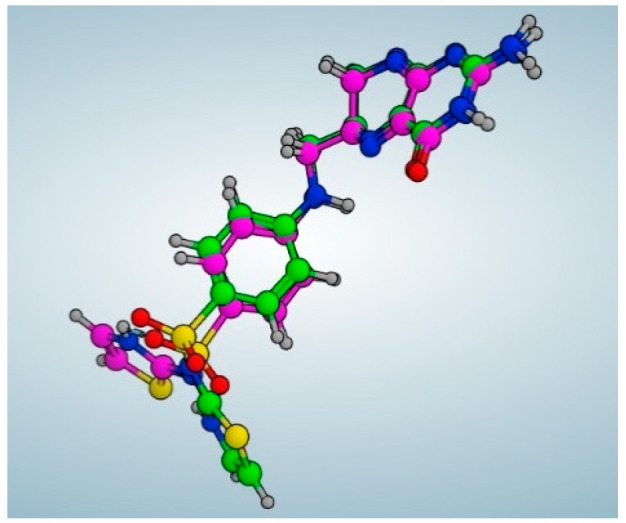
Redocking of STZ (purple) and its superimposition on the co-crystallized STZ (green).

Redocking STZ into the DHPS substrate-binding site was performed through the rigid docking protocol. Findings from the STZ redocking analysis revealed a binding score of (−6.678 kcal/mol) and an RMSD of (1.1240 Å) relative to the co-crystalline ligand; this indicates a well-matched superimposition (Figure 16). The accepted docking technique was proven to be legitimate because it displayed RMSD values below 2.0, and these validation findings demonstrated that the selected docking parameters and methodologies were the best for choosing the ideal docking pose.

**Figure 17:**
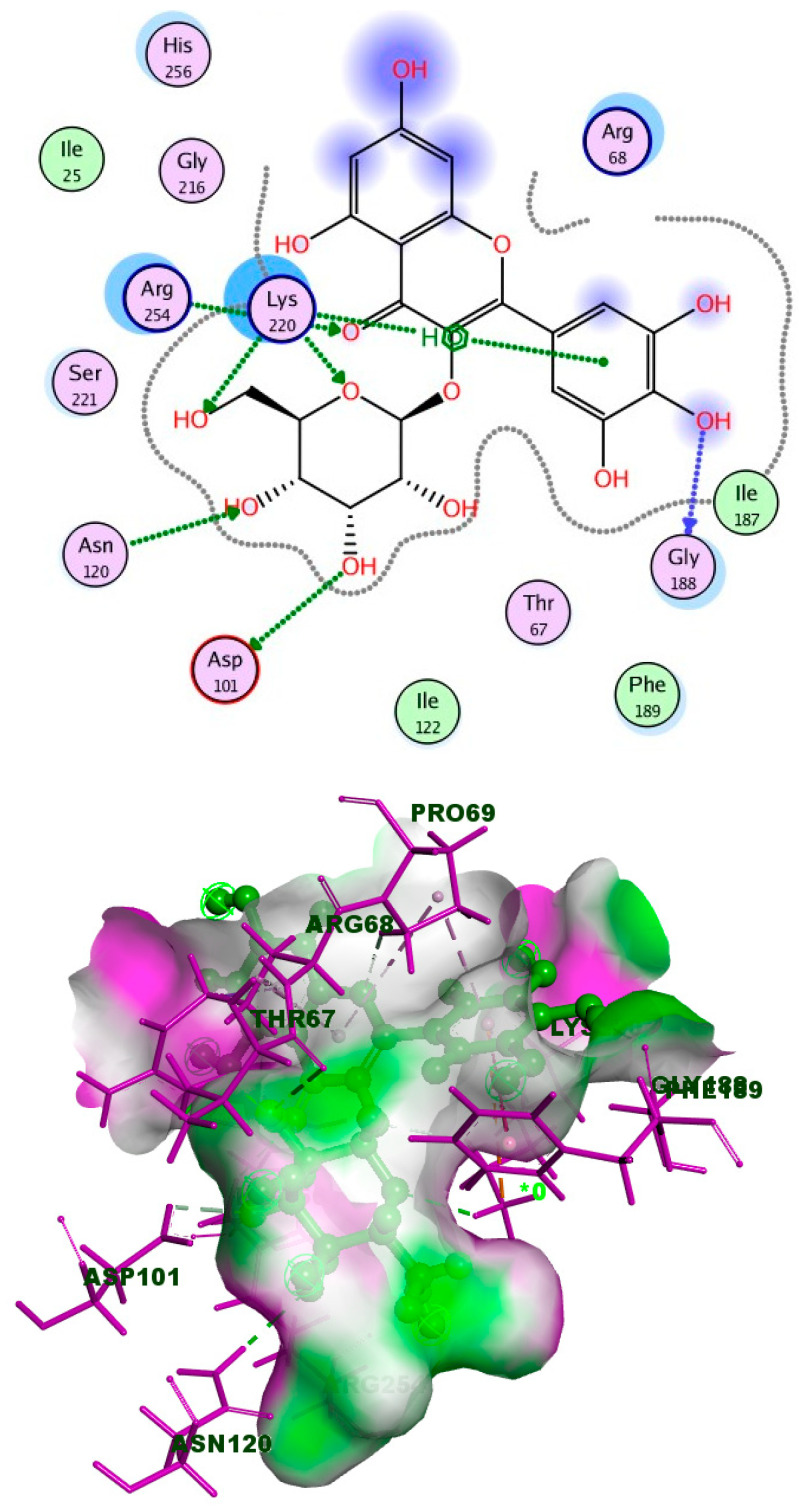
Myricetin-3-O-glucoside’s 2D and 3D interactions with DHPS.

It is obvious from the results of docking that myricetin-3-glucoside had the best binding energy (−10.574 kcal/mol), a value which exceeded that of the co-crystallized ligand (STZ), which showed a binding energy of (−6.678 kcal/mol). The 2D and 3D interactions of myricetin-3-glucoside with the DHPS enzyme are depicted in Figure 17.

**Figure 18:**
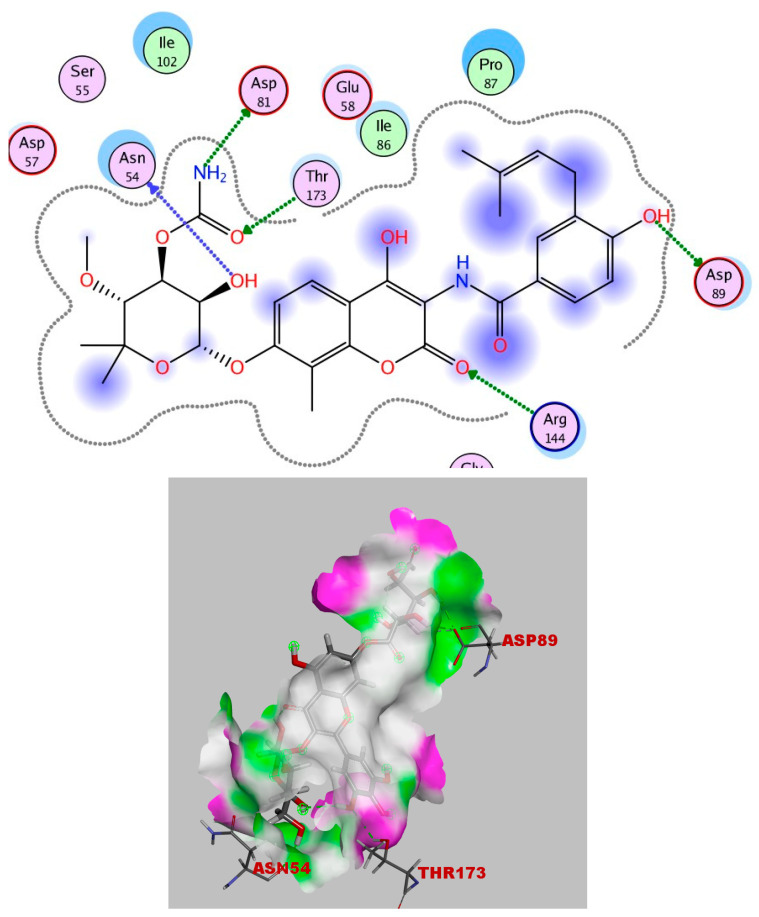
Novobiocin’s 2D and 3D interactions with DNA gyrase.

Furthermore, the crystal structure of Staph Gyrase B 24kDa in complex with Novobiocin was downloaded from the protein data bank (PDB ID: 4uro) www.rcsb.org (accessed on 15 September 2023). In this study, the co-crystallized ligand (Novobiocin) was re-docked to the active site DNA-gyrase to, at first, confirm the molecular docking technique. Novobiocin’s validation results revealed a score energy of −9.308 kcal/mol and an RMSD of 0.954 Å. The hydrogen bonds formed by Asp81, Asp89, Asn54, Arg144, and Thr173 in the docking position allowed for interactions with the important amino acids in the active site (Figure 18).

**Figure 19:**
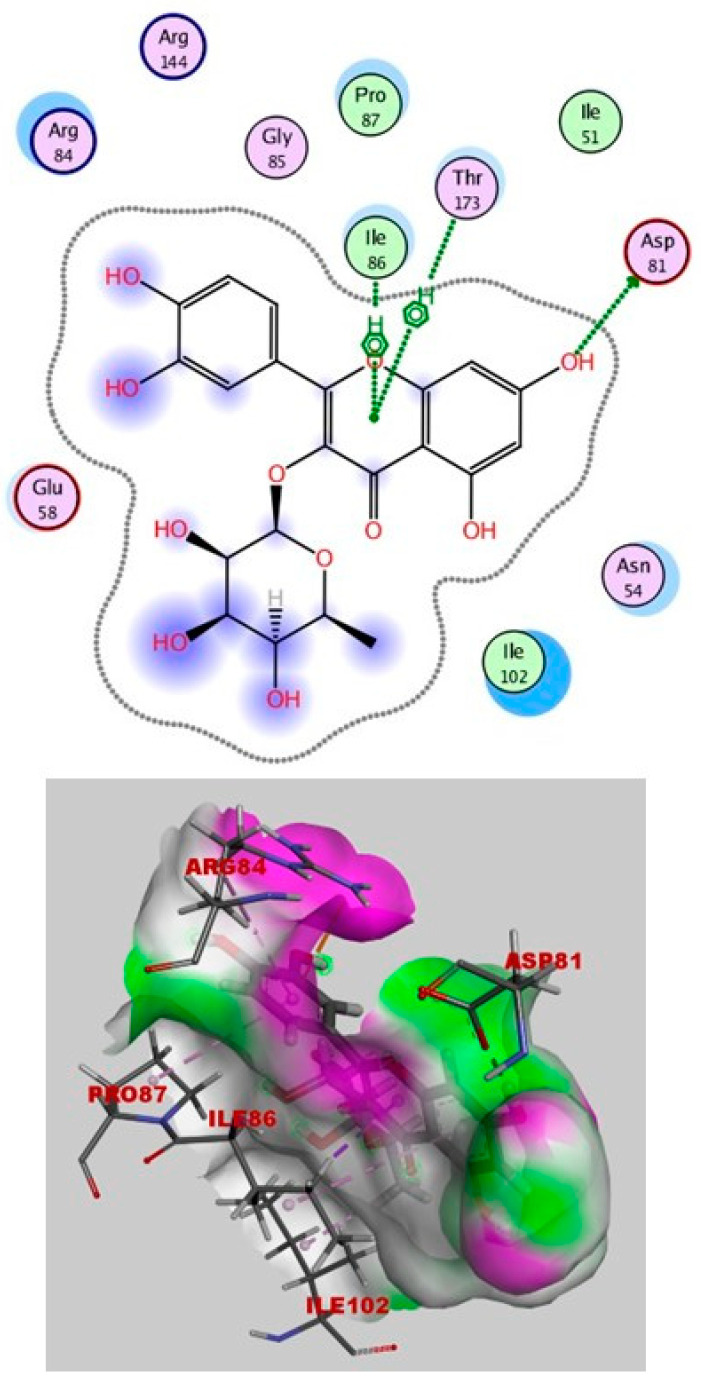
Quercetin rhamnoside’s 2D and 3D interactions with DNA gyrase.

It is obvious from the results of docking that quercetin rhamnoside had the best binding energy (−8.738 kcal/mol). In Figure 19, the 2D and 3D analyses of quercetin rhamnoside with the DNA gyrase enzyme are displayed.

## 3. Experimental Design

### 3.1. Plant Material

The non-flowering aerial parts of the *Aeonium arboreum* variety *atropurpureum* (purple) were collected in December 2018 from Saft El Laban, Giza Governorate, Egypt. A voucher specimen (Sp. No. 26.6.23) was stored at Cairo University’s Faculty of Pharmacy herbarium. This specimen was authenticated by Dr. Reem Samir Hamdy, Professor at the Faculty of Science, Botany Department. To remove any dust or impurities, the collected aerial parts were rinsed with tap water, and then dried in the shade for three weeks until completely dry. Then, the dried aerial parts were gathered and ground into a powder to prepare them for extraction.

### 3.2. Chemicals

The utilized reagents were of analytical grade. Chemicals were purchased from the biochem Company and the Al-Nasr Company for chemical industries in el-ameerya.

### 3.3. Extraction Procedure

*A. arboreum*’s non-flowering aerial parts were air-dried and ground, and 1.5 kg of that material was extracted with methanol (MeOH), using Ultraturrax, and at room temperature, until exhaustion. The extract was filtered and concentrated at 45 °C under decreased pressure via a rotary evaporator to produce a dark greenish-brown residue (257 g).

### 3.4. Metabolic Profiling of Aeonium arboreum Methanolic Crude Extract

*A. arboreum* methanolic extract was subjected to LC-MS profiling using Acquity liquid chromatography coupled to a Synapt G2 HDMS quadrupole time-of-flight hybrid mass spectrometer (Waters, Milford, CT, USA), following the procedure outlined by Haggag et al. [28], to provide broad qualitative profiles of metabolites that might be indicators in the activity of the extracts [29]. Dereplication is the process of quick analysis and quantification of known secondary metabolites in unfractionated crude extracts [30]. A BEH C18 column (2.1 × 10^2^ mm, 1.7 mm particle size, Waters, Milford, CT, USA) and guard column (2.1 × 5 mm, 1.7 mm particle size) were used for chromatographic separation. A linear binary solvent gradient of acetonitrile as solvent B and 0.1% formic acid in water as solvent A was carried out over six minutes at a flow rate of 0.3 mL per minute at 40 °C; the injection volume was 2 µL. A Thermo Xcalibur 2.1 (Thermo Scientific, Germany) was used to view LC-MS spectra. The raw data were transformed using MSConvert software (version 1) into distinct positive and negative ionization files. Peak selection, deconvolution, deisotoping, alignment, and formula prediction were all performed using MZmine 2.10 software. The Dictionary of Natural Products Database (DNP) and the METLIN database were utilized for the compounds’ identification.

### 3.5. Green Synthesis of Silver Nanoparticles

At room temperature, AgNPs were created by reducing 10 mL of a constant concentration (1 mM) AgNO_3_ solution with varying extract quantities (100 μL to 500 μL) from the stock solution (0.4192 g extract/10 mL solvent). After giving the prepared blend a shaking by hand, it was left to stand in the dark for two hours. After repeatedly centrifuging the produced AgNPs for 20 min at 10,000 rpm, they were redispersed in deionized water to purify them. To remove unwanted materials and separate the pure AgNPs, this procedure was performed twice [31,32,33].

### 3.6. Characterization of Silver Nanoparticles

#### 3.6.1. UV Spectral Analysis

Determination of the band metal wavelength was performed using the V-730 UV-Vis spectrophotometer (Jasco Co., Tokyo, Japan).

#### 3.6.2. FTIR Analysis

Using an FTIR (Fourier transform infrared) spectrometer (VERTEX 80v, Bruker, Billerica, MA, USA), the chemical composition of the synthesized sample was recorded.

#### 3.6.3. Zeta-Sizer Measurements

The particle size analyzer (Nano-ZS, Malvern Instruments Ltd., Malvern, UK) was used to measure the samples’ average diameter, size distribution, and Zeta potential. To guarantee an appropriate scattering intensity, the material was sonicated for ten to twenty minutes before testing.

#### 3.6.4. Transmission Electron Microscopy (TEM)

The size and distribution of the synthesized samples were assessed using transmission electron microscopy (TEM; JEOL, JEM-1011; Tokyo, Japan).

#### 3.6.5. X-ray Diffraction (XRD)

A Bruker D8 Discover Diffractometer (Karlsruhe, Germany) was utilized with a Cu target, using wavelength 1.54 A, 40 kv, 40 mA.

### 3.7. Antimicrobial Activity

#### 3.7.1. Determination of Antimicrobial Activity

Antimicrobial evaluations of the methanolic extract of *A. arboreum* and its nanoform product (AgNPs) were determined using an agar well diffusion assay, according to the method described by Hamed et al. [34]. Five standard strains of bacteria and yeast described as causing urinary tract infections were used in the evaluation test. The strains of *Staphylococcus aureus* (ATCC 6538), *Escherichia coli* (ATCC 8739), *Enterococcus faecalis* (*ATCC 10541*), *Pseudomonas aeruginosa* (ATCC 90274), and *Candida albicans* (ATCC 10221) were obtained from VACSERA (the Egyptian Company for Biological Products and Vaccines), Giza, Egypt. Briefly, plates of Muller–Hinton agar (MHA) (Oxoid, Hampshire, UK) were inoculated with 1.8×10^8^ CFU/mL of the tested strains. Wells of 6 mm diameter were punched with a sterile cork-borer, and then 100 μL of each test sample, at a concentration of 10 mg/mL, and dissolved in 5% DMSO, was introduced into the wells. Blanks (containing only solvent) and standard antibiotic (gentamycin) were also tested against each strain. For 24 h, the agar plates were incubated at 37 °C. Following incubation, the inhibitory zones generated were measured in millimeters, in accordance with CLSI recommendations.

#### 3.7.2. Determination of Minimum Inhibitory Concentration (MIC) and Minimum Bactericidal Concentration (MBC)

The MICs of the *A. arboreum* extract and the AgNPs were determined against the five tested strains using the broth microdilution method. Briefly, stock solutions were prepared for the test samples by dissolving them in DMSO (10% of the final volume) and diluting the result with culture broth to a concentration of 1 mg/mL. Further two-fold serial dilutions were performed, reaching concentrations ranging from 1000 μg/mL to 100 μL for each dilution. The prepared dilutions of extract and AgNPs were distributed in 96-well plates. Wells were inoculated with 5 μL of bacterial suspension (5 × 10^5^ CFU/well) and incubated at 37 °C for 24 h. Wells of positive controls (containing media and bacteria, and without antimicrobials) and negative controls (containing only culture broth, without bacteria) were also prepared. The absorbance of bacterial growth was measured at 600 nm using a microplate reader (Infinite M1000 Pro, Tecan Company, Switzerland). The values of MIC were recorded as the minimum concentration of extract that inhibited visible growth [35].

A volume of 10 μL was taken from each test sample; these exhibited no evident growth after 24 h incubation, and were subsequently inoculated onto agar plates. These plates were kept at 37 °C for another 24 h. The MBC was defined as the lowest drug concentration which demonstrates reduction of bacterial growth by 99.9% [36]. When the equivalent MBC/MIC ratio is less than 4, the extract is thought to have bactericidal activity; nevertheless, when it is greater than 8, the extract is thought to have bacteriostatic properties [37].

#### 3.7.3. Determination of Antibiofilm Activity

Biofilm-inhibiting activity of *A. arboreum* extract and AgNPs was evaluated in vitro against the bacterial strains and candida, using a Microplate Dilution assay according to the technique described in Wu et al. [38]. Briefly, a 10 μg portion of bacteria cell suspension (1 × 10^5^ CFU/mL) was inoculated, with 190l TSB medium supplemented with 1% glucose, in each well of pre-sterilized 96-well flat-bottom polystyrene microtiter plates. Gradient subinhibitory concentrations of extracts at 1/4, 1/2, and 3/4 MIC were applied into wells incubated for 24 h at 37 °C. Plates were washed twice with phosphate buffer saline (PBS) and left to dry. A volume of 200 μL of crystal violet solution (0.2%) was added to each well. After 15 min, the excess crystal violet was removed, and the cell-bound crystal violet was dissolved in 33% acetic acid. The absorbance was measured at O.D 600 nm using a Spectrostar Nano Microplate Reader (BMG LABTECH GmbH, Allmendgrun, Germany). The biofilm inhibition ability of the sample was calculated as follows:

(1) $$\begin{array}{c} Biofilm\;inhibition\;ability=[(absorbance\;control-absorbance\;Blank)\;-\\ (absorbance\;sample-absorbance\;Blank)/(absorbance\;control-absorbance\\ Blank)]\times 100 \end{array}$$

#### 3.7.4. Determination of the Antihemolytic Activity of Plant Extract and Nano-Formula

The total extract of *A. arboreum* and AgNPs were evaluated for their capacity to inhibit the hemolysis of blood erythrocyte cells produced by the tested bacterial strains. Fresh microbial suspensions were prepared and adjusted to an OD 600, and then centrifuged at 21 × 10^3^× *g* for 20 min. A volume of 500 μL of each produced supernatant was added to subinhibitory concentrations of both the extract and the AgNPs (50% and 75% of MIC), treated with 300 μL of 2% fresh sterile erythrocytes suspension to compose final volumes for each of 1 mL. The erythrocyte suspension was treated with 0.1% sodium dodecyl sulphate to create a positive control (hemolyzed), and LB broth was used to incubate erythrocytes under the same conditions to create a negative control (unhemolyzed). A UV spectrophotometer was used to measure the absorbance of each solution at 530 nm, after each tube had been incubated for four hours at 37 °C and centrifuged for ten minutes at 4000 rpm [39].

The following formula was used to determine the percentage of hemolysis:

(2) $$\begin{array}{c} {}[(Sample\;with\;bacterial\;culture-Negative\;control)/(Positive\;control\;-\\ Negative\;control)]\times 100 \end{array}$$

### 3.8. Molecular Docking

#### 3.8.1. Simulations of Molecular Docking

The synthesis of each of the proteins and the ligand, molecular docking, and evaluation of the ligand–protein interaction using the scoring function and visualization of poses were all accomplished using the molecular operating environment (MOE) 2019.0102 [40]. The specifications were as follows: Utilized docking placement, Triangular matcher; rescoring, London dG; forcefield and refinement, Affinity dG; and the docking procedure was carried out via the Amber10 protocol.

#### 3.8.2. Target Protein Structure Preparation

Dihydrofolate reductase (DHFR), dihydropteroate synthase (DHPS), and DNA gyrase were retrieved from the Protein Data Bank www.rcsb.org (accessed on 15 September 2023) with PDB id: 3frb, 3tye, and 4uro, respectively. The protein structure was analyzed and fixed using MOE’s automatic correction and fixing order. The structure was augmented with hydrogen atoms during the protonation phase. Using scoring values and visualized poses, findings were viewed and filtered when docking was finished.

#### 3.8.3. Investigated Drug Molecules’ Preparation

MOE was used to create a 3D model library from the active and selective target drugs. This chemical underwent an energy minimization method and automatic partial charge computation. It was ultimately saved as an MDB file for use in the docking calculations with the target enzyme.

#### 3.8.4. Docking Validation

To verify the docking procedure, the root mean square deviation, or RMSD, is computed. The RMSD is projected by redocking the co-crystallized ligand on its target enzyme, then superimposing it on its original co-crystallized restricted conformation.

## 4. Conclusions

This study successfully demonstrated, for the first time, biosynthesis and characterization of silver nanoparticles (AgNPs) using *Aeonium arboreum* extract. UPLC-Q/TOF-MS analysis identified 11 potential bioactive metabolites in the extract. The synthesized AgNPs exhibited superior antibacterial, antibiofilm, and antihemolytic activities against various uropathogens (*S. aureus*, *E. coli*, *E. faecalis*, *P. aeruginosa*, and *C. albicans*), compared to the *A. arboreum* extract alone. Notably, the AgNPs displayed minimum inhibitory concentrations (MICs) similar to or lower than gentamicin, a commonly used antibiotic, suggesting the former’s potential efficacy against urinary tract infections (UTIs). In silico docking studies further supported this potential by revealing promising binding affinities of key metabolites (myricetin-3-O-glucoside and quercetin-3-O-rhamnoside) to enzymes crucial for bacterial survival (DHFR, DHPS, and DNA gyrase). These findings highlight the promise of *A. arboreum* as a source of potent antibacterial metabolites and AgNPs compounds with potential applications in UTI treatment, including possible use as a natural substitute for sulfa drugs. Yet further in-depth HPLC qualitative and quantitative characterization of the plant’s active principles is recommended in order to gain a deeper understanding of the plant’s active ingredients; this may potentially lead to the identification of additional, intriguing bioactive components. Moreover, additional comparative studies would be beneficial in thoroughly assessing the nanoparticles’ relative effectiveness in comparison to other *Aeonium arboreum* extract forms and isolated compounds, alone, and in combination with nanoparticles

Future research should focus on elucidating the detailed mechanisms underlying the antibacterial activity of both the *A. arboreum* extract and the biogenic AgNPs, as well as conducting comprehensive in vivo pharmacological evaluations of AgNPs, as compared to the original extract, to assess their efficacy and safety before clinical trials. It would also be useful to reveal the effects of *A. arboreum* metabolites on the biosynthesized AgNPs’ physicochemical parameters in order to optimize the biological activities of the latter. Additionally, these biogenic AgNPs should undergo additional toxicity studies, assessing compatibility with biological environments, as well as stability studies designed to offer a more thorough comprehension of the silver nanoparticles’ safety and suitability for prospective biomedical uses.

## Figures and Tables

**Table 1:** Zones of inhibition of *A. arboreum* and nanoform (AgNPs) against the tested bacterial strains.

Test Strains		Zone of Inhibition (mm)		
		*A. arboreum*	AgNPs	Control (Gentamycin)
Gram-positive	*Staphylococcus. aureus* (ATCC 6538)	23 ± 0.2	25 ± 0.1	22 ± 0.3
	*Enterococcus faecalis* (ATCC 10541)	27 ± 0.1	30 ± 0.1	28 ± 0.1
Gram-negative	*Escherichia coli* (ATCC 8739)	27 ± 0.1	30 ± 0.3	24 ± 0.2
	*Pseudomonas aeruginosa* (ATCC 90274)	17 ± 0.2	22 ± 0.1	18 ± 0.1
Yeast	*Candida albicans* (ATCC 10221)	28 ± 0.3	33 ± 0.1	27 ± 0.3

**Table 2:** The MBC/MIC ratios of *A. arboreum* extract and AgNPs against standard tested strains.

Test Strains	*A. arboreum*			AgNPs			Control (Gentamycin)	
	MIC μg/mL	MBC μg/mL	MBC/MIC	MIC μg/mL	MBC μg/mL	MBC/MIC	MIC μg/mL	MBC μg/mL
*Staphylococcus aureus*	15.62	31.25	2	7.8	15.62	2	15.62	15.62
*Enterococcus faecalis*	7.8	15.62	2	3.9	7.8	2	7.8	7.8
*Escherichia coli*	15.62	15.62	1	7.8	7.8	1	31.25	31.25
*Pseudomonas aeruginosa*	62.5	250	4	31.25	31.25	1	31.25	62.5
*Candida albicans*	31.25	62.5	2	15.62	15.62	1	15.62	31.25

Extract concentration = 10 mg/mL, cup diameter = 6 mm.

## Data Availability

Data will be available on request from the corresponding author.

## Supplementary Materials

The following supporting information can be downloaded at: https://www.mdpi.com/article/10.3390/ijms25158039/s1. References [22,41,42,43,44,45,46,47,48,49,50,51,52] belongs to data in Supplementary File.
