# Supplementary material for: Biosynthesis and Characterization of Aeonium arboreum-Derived Silver Nanoparticles: Antimicrobial Activity, Biofilm Inhibition, Antihemolytic Activity, and In Silico Studies
Source: Int J Mol Sci. 2024 Jul 23;25(15):8039. doi: 10.3390/ijms25158039 (PMC11312205; doi:10.3390/ijms25158039)
Supplement: Supplementary file 1 [file ijms-25-08039-s001.zip › ijms-3097690-supplementary.pdf]

Biosynthesis and Characterization of *Aeonium arboreum*-Derived Silver Nanoparticles: Antimicrobial Activity, Biofilm Inhibition, Antihemolytic Activity, and In Silico Studies

Marwah M. Alfeqy[1*], Seham S. Elhawary[2], Ali M. El-Halawany[2], Mohamed A. Rabeh[3], Saad A. Alshehri[3], Usama Ramadan Abdelmohsen[4,5], Nesreen A.Safwat[6], Aya M. Serry[7], Heba A. Fahmy[1] †, Marwa I. Ezzat[2*]†

[1]Pharmacognosy Department, Faculty of Pharmacy, Modern University for Technology & Information, Cairo, Egypt
[2]Pharmacognosy Department, Faculty of Pharmacy, Cairo University, Kasr El Aini 11562, Cairo, Egypt
[3]Pharmacognosy Department, College of Pharmacy, King Khalid University, Abha 62251, Saudi Arabia
[4]Pharmacognosy Department, Faculty of Pharmacy, Minia University, Minia, Egypt.
[5]Pharmacognosy Department, Faculty of Pharmacy, Deraya University, New Minia, Egypt.
[6]Microbiology & Immunology Department, Faculty of Pharmacy, Modern University for Technology & Information, Cairo, Egypt
[7]Pharmaceutical chemistry Department, Faculty of Pharmacy, Modern University for Technology & Information, Cairo, Egypt

Corresponding authors:
*Corresponding author: M. I. Ezzat, marwa.ezzat@pharma.cu.edu.eg
**Co-corresponding author: Marwah M. Alfeqy, drmarwahalfeqy@gmail.com
†: the last two authors have equal contributions

Supplementary data

Table S1. Tentatively identified Secondary Metabolites from methanol extract of *A.arboreum* aerial parts

Peak no	Retention time (min)	m/z		M. wt.	Name	Molecular formula	References
		[M-H]+	[M-H]-				
1	0.7347611		160.8406	161.84787	1,1,2-Ethanetricarboxylic acid	$C_5H_6O_6$	[1, 2]
2	0.8920111		169.01286	170.02013	Gallic acid	$C_7H_6O_5$	[3]
3	2.8351667		479.08223	480.08951	Myricetin 3-glucoside	$C_{21}H_{20}O_{13}$	[4-6]
4	3.0440611		463.08697	464.09425	Quercetin-3-*O*-glucoside	$C_{21}H_{20}O_{12}$	[7]
5	3.1283278		163.0386	164.04587	*P*-Coumaric acid	$C_9H_8O_3$	[8]
6	3.3524556		447.09228	448.09955	Quercetin-3-*O*-rhamnoside	$C_{21}H_{20}O_{11}$	[9]
7	3.7195111	181.04763		180.04035	Caffeic acid	$C_9H_8O_4$	[10]
8	4.5083889		301.03408	302.04135	Quercetin	$C_{15}H_{10}O_7$	[3, 6]
9	5.0713833		329.06578	330.07305	Isoguarabin	$C_{17}H_{14}O_7$	[11]
10	8.7831944		377.16052	378.1678	Daphneresinol	$C_{20}H_{26}O_7$	[12]
11	10.518528	623.82155		622.81427	Dammar-24-ene-3,12,20-triol,3-O-Glucopyranoside	$C_{38}H_{68}O_8$	[13]

Table S2: Docking score results of the extract components of *A.arboreum* on DHFR

Compound	S-score (kcal/mol)	Involved receptor residues	Bond length (Å)	Type of bond interaction
**Quercetin glucoside**	-8.908	ASN 18<br>ILE 14<br>SER 49	2.93<br>2.86<br>3.08	H-bond
**Quercetin Rhamnoside**	-10.047	ASP 27<br>ASP 27<br>LEU 5<br>ASN 18	2.80 3.00<br>3.02<br>3.10	H-bond
		ILE 50	3.78	pi-H
**Quercetin**	-4.315	GLN 95	3.12	H-bond
**Myricetin-3-glucoside**	-10.723	GLN 19<br>ASP 27<br>PHE 92	3.02<br>3.28<br>3.15	H-bond<br>H-bond Backbone donor
***P*-Coumaric acid**	-4.248	SER 49<br>PHE 92	3.08<br>2.92	H-bond
**Gallic acid**	-2.965	THR 46<br>LYS 45	2.88<br>2.95	H-bond
		LYS 45	2.95	Ionic

		LYS 45	3.95	
**Daphneresinol**	-4.264	GLU 100<br>ARG 44<br>SER 64	2.80<br>2.95<br>3.37	H-bond
		ARG 44	2.95	Ionic
**Dammar-24-ene-3,12,20-triol,3-O-Glucopyranoside**	-5.910	SER 49<br>LYS 45	2.79<br>2.95	H-bond
**Caffeic acid**	-4.556	ILE 14	2.93	H-bond
**1,1,2-Ethanetricarboxylic acid**	-3.354	VAL 6<br>ALA 7	3.39<br>3.09	H-bond
**Isoguarabin**	-3.775	HIS 77	2.96	H-bond

Table S3: Docking score results of the extract components of *A.arboreum* on DHPS

Compound	S-score (kcal/mol)	Involved receptor residues	Bond length (Å)	Type of bond interaction
**Quercetin glucoside**	-9.925	ASP 101<br>GLY 188<br>ARG 68	2.73<br>3.04<br>2.80	H-bond
**Quercetin Rhamnoside**	-9.756	MET 145<br>ASP 101<br>GLY 188<br>ARG 68	4.05<br>2.74<br>2.97<br>2.86	H-bond
**Quercetin**	-7.802	ASP 101<br>GLY 188<br>ARG 68	2.71<br>3.10<br>2.82	H-bond
**Myricetin-3-glucoside**	-10.574	GLY 188<br>ASP 101<br>ARG 254<br>ARG 254<br>LYS 220<br>LYS 220<br>ASN 120	2.84<br>3.43<br>3.07<br>3.44<br>3.29<br>3.36<br>3.29	H-bond
		LYS 220	4.10	pi-H
***P*-Coumaric acid**	-5.414	ARG 68<br>ARG 254<br>ARG 254	3.00<br>3.13<br>3.09	H-bond
**Gallic acid**	-5.944	SP 184<br>ASP 101<br>ARG 68<br>ARG 254<br>ARG 68<br>ARG 254	3.48<br>2.87<br>3.04<br>3.02<br>3.04<br>3.27	H-bond
		ARG 254<br>ARG 254	3.02<br>3.96	Ionic
**Daphneresinol**	-9.320	SER 221<br>ARG 68<br>ARG 68	3.13<br>3.42<br>2.85	H-bond
		ARG 68	2.85	Ionic
**Dammar-24-ene-3,12,20-triol,3-O-Glucopyranoside**	-8.922	ARG 234	3.08	H-bond
**Caffeic acid**	-5.510	SP 101<br>ASP 184<br>ARG 68	2.94<br>3.37<br>3.10	H-bond
		PHE 189	4.49	pi-H
**1,1,2-Ethanetricarboxylic acid**	-5.438	MET 145<br>MET 145	4.08<br>3.12	H-bond

		MET 145<br>LYS 220	3.42<br>3.04	
**Isoguarabin**	-7.350	ASP 184<br>SER 218	2.68<br>2.80	H-bond

Table S4: Docking score results of the extract components of *A.arboreum* on DNA gyrase

Compound	S-score (kcal/mol)	Involved receptor residues	Bond length (Å)	Type of bond interaction
**Quercetin glucoside**	-8.727	SER 128	2.91	H-bond
**Quercetin Rhamnoside**	-8.738	ASP 81	2.82	H-bond
		ILE 86<br>THR 173	3.85<br>4.37	Pi-H
**Quercetin**	-7.224	GLU 58<br>GLY 85	2.77<br>3.22	H-bond
**Myricetin-3-glucoside**	-8.537	ASP 57<br>ARG 84	3.07<br>3.04	H-bond
***P*-Coumaric acid**	-5.799	GLU 58	3.20	H-bond
		THR 173	3.62	Pi-H
**Gallic acid**	-5.434	ASP 81	3.11	H-bond
		THR 173	3.54	Pi-H
**Daphneresinol**	-8.520	GLU 58	2.72	H-bond
		THR 173	4.11	Pi-H
**Dammar-24-ene-3,12,20-triol, 3-O-Glucopyranoside**	-8.027	GLU 58<br>ARG 84	2.86<br>3.33	H-bond
**Caffeic acid**	-6.527	ASP 81	2.90	H-bond
**1,1,2-Ethanetricarboxylic acid**	-5.233	GLU 58<br>ASN 54	2.89<br>3.10	H-bond
**Isoguarabin**	-7.327	THR 173	2.88	H-bond

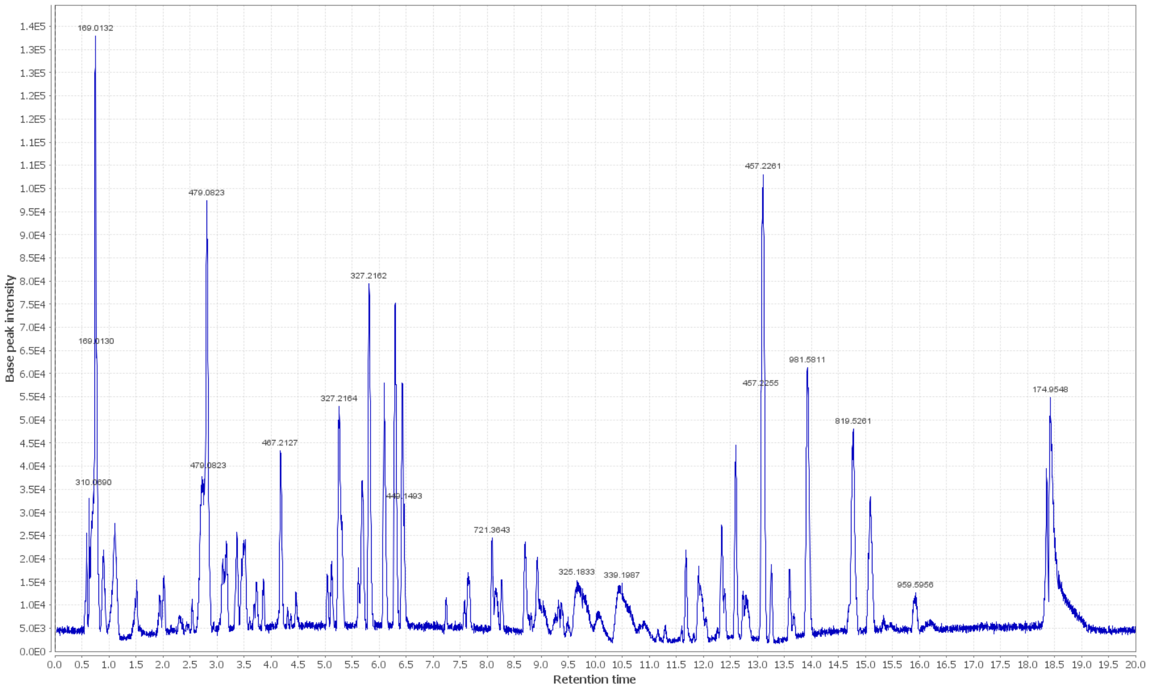

Figure (S1a). Total ion chromatogram of *A.arboreum* methanolic extract in negative ionization mode using UPLC-Q/TOF-MS

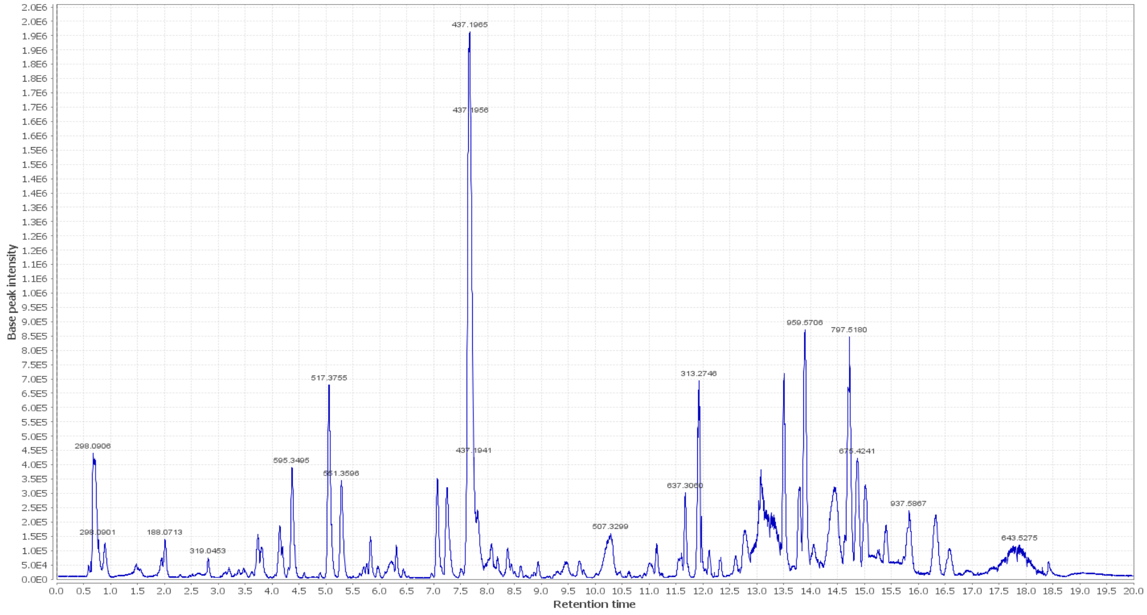

Figure (S1b). Total ion chromatogram of *A.arboreum* methanolic extract in Positive ionization mode using UPLC-Q/TOF-MS

1. Bylund, D., et al., *Analysis of low molecular mass organic acids in natural waters by ion exclusion chromatography tandem mass spectrometry.* Journal of Chromatography A, 2007. **1176**(1-2): p. 89-93.
2. Affes, S., et al., *ESI-MS/MS analysis of phenolic compounds from Aeonium arboreum leaf extracts and evaluation of their antioxidant and antimicrobial activities.* Molecules, 2021. **26**(14): p. 4338.
3. Fang, Z., M. Zhang, and L. Wang, *HPLC-DAD-ESIMS analysis of phenolic compounds in bayberries (Myrica rubra Sieb. et Zucc.).* Food Chemistry, 2007. **100**(2): p. 845-852.
4. Milad, R., S. El-Ahmady, and A.N. Singab, *Genus Kalanchoe (Crassulaceae): A review of its ethnomedicinal, botanical, chemical and pharmacological properties.* European Journal of Medicinal Plants, 2014. **4**(1): p. 86.
5. Bensouici, C., et al., *Compounds from Sedum caeruleum with antioxidant, anticholinesterase, and antibacterial activities.* Pharmaceutical biology, 2016. **54**(1): p. 174-179.
6. Gaind, K., A.K. Singla, and J.W. Wallace, *Flavonoid glycosides of Kalanchoe spathulata.* Phytochemistry, 1981. **20**(3): p. 530-531.
7. Ablajan, K., et al., *Structural characterization of flavonol 3, 7 ‐di ‐O ‐glycosides and determination of the glycosylation position by using negative ion electrospray ionization tandem mass spectrometry.* Journal of Mass Spectrometry, 2006. **41**(3): p. 352-360.
8. Mateos, R., et al., *Improved LC-MSn characterization of hydroxycinnamic acid derivatives and flavonols in different commercial mate (Ilex paraguariensis) brands. Quantification of polyphenols, methylxanthines, and antioxidant activity.* Food Chemistry, 2018. **241**: p. 232-241.
9. Dewi, R.T., et al. *Bioconversion of quercetin glucosides from Dendrophthoe pentandra leaf using Aspergillus acueletus LS04-3*. in *AIP Conference Proceedings*. 2019. AIP Publishing.
10. Lin, Y., et al., *Qualitative and quantitative analysis of phenolic acids, flavonoids and iridoid glycosides in Yinhua Kanggan tablet by UPLC-QqQ-MS/MS.* Molecules, 2015. **20**(7): p. 12209-12228.
11. 杜玖珍, 秦民坚, and 须春君, *盾木酮类化合物在植物中的分布及其结构研究进展.* 现代药物与临床, 2011. **26**(3): p. 174-180.
12. Liang, S., et al., *Phenylpropanoids from Daphne feddei and their inhibitory activities against NO production.* Journal of natural products, 2008. **71**(11): p. 1902-1905.
13. Jiang, Y., H. Gao, and G. Turdu, *Traditional Chinese medicinal herbs as potential AChE inhibitors for anti-Alzheimer's disease: A review.* Bioorganic chemistry, 2017. **75**: p. 50-61.
